# Genomic findings in schizophrenia and their implications

**DOI:** 10.1038/s41380-023-02293-8

**Published:** 2023-10-18

**Authors:** Michael J. Owen, Sophie E. Legge, Elliott Rees, James T. R. Walters, Michael C. O’Donovan

**Affiliations:** Centre for Neuropsychiatric Genetics and Genomics, Division of Psychological Medicine and Clinical Neurosciences, Cardiff University, Cardiff, UK

**Keywords:** Schizophrenia, Genetics

## Abstract

There has been substantial progress in understanding the genetics of schizophrenia over the past 15 years. This has revealed a highly polygenic condition with the majority of the currently explained heritability coming from common alleles of small effect but with additional contributions from rare copy number and coding variants. Many specific genes and loci have been implicated that provide a firm basis upon which mechanistic research can proceed. These point to disturbances in neuronal, and particularly synaptic, functions that are not confined to a small number of brain regions and circuits. Genetic findings have also revealed the nature of schizophrenia’s close relationship to other conditions, particularly bipolar disorder and childhood neurodevelopmental disorders, and provided an explanation for how common risk alleles persist in the population in the face of reduced fecundity. Current genomic approaches only potentially explain around 40% of heritability, but only a small proportion of this is attributable to robustly identified loci. The extreme polygenicity poses challenges for understanding biological mechanisms. The high degree of pleiotropy points to the need for more transdiagnostic research and the shortcomings of current diagnostic criteria as means of delineating biologically distinct strata. It also poses challenges for inferring causality in observational and experimental studies in both humans and model systems. Finally, the Eurocentric bias of genomic studies needs to be rectified to maximise benefits and ensure these are felt across diverse communities. Further advances are likely to come through the application of new and emerging technologies, such as whole-genome and long-read sequencing, to large and diverse samples. Substantive progress in biological understanding will require parallel advances in functional genomics and proteomics applied to the brain across developmental stages. For these efforts to succeed in identifying disease mechanisms and defining novel strata they will need to be combined with sufficiently granular phenotypic data.

## Introduction

Schizophrenia is a highly heritable psychiatric condition with a lifetime prevalence of around 1%. It is a highly complex, multi-domain syndrome which is associated with perturbations in many aspects of brain function [1]. Its core features, around which modern diagnostic criteria have been built, consist of a combination of positive, negative and disorganised symptoms as well as certain exclusion criteria [2, 3]. However, these central attributes are frequently accompanied by a wide range of other features, including impairments of most aspects of cognitive function [4], affective symptoms [3], movement disorders [5] and sensory abnormalities [6]. Among those who meet diagnostic criteria, there is considerable heterogeneity in individual symptoms, mode of onset, course, and outcome [3, 7]. The boundaries between schizophrenia and other psychiatric syndromes are indistinct, as are the boundaries with wellness [3]. For instance, there is overlap in symptoms with schizoaffective disorder, bipolar disorder and childhood neurodevelopmental disorders [8, 9]. Antipsychotic drugs form the mainstay of current pharmacotherapy, but these are largely ineffective in treating negative and disorganised symptoms, are ineffective in treating psychosis in around 30% of cases and are associated with a significant number of adverse effects [3]. Therapeutic advances are badly needed, but these have proved elusive with progress impeded by a poor understanding of pathophysiology, clinical heterogeneity and a lack of valid biomarkers and model systems [10].

The high heritability of schizophrenia together with advances in genomic technology and the complexity and inaccessibility of the human brain have driven a substantial effort to understand the genetics of the condition in the hope that this will illuminate pathogenesis and provide novel approaches to prediction and stratification. This intense and highly collaborative endeavour has, over the past 15 years, resulted in considerable progress. In this article, we will review recent findings, summarise the key insights these have revealed, and consider important remaining challenges and how these can be met.

### Genetic architecture

#### Common variants

**Fig. 1:**
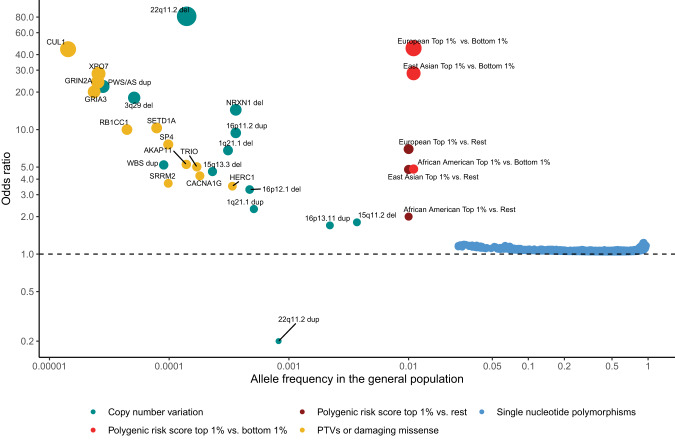
Effect size and frequency of known risk variants for schizophrenia. Effect sizes expressed as odds ratios (OR) versus general population frequencies for copy number variants (CNVs), damaging rare coding variants (protein-truncating variants (PTVs) or missense variants), common single nucleotide polymorphisms (SNPs), and polygenic risk scores (PRS). The OR less than 1 of 22q11dup CNV denotes a protective effect. CNVs effect sizes are from [20]. To constrain the ORs for 22q11.2 del and PWS/AS dup below infinity, a single carrier for each CNV was added to the controls. Dup and del refer to duplication and deletions. PTV and missense associated genes are from references [29, 30]. Population frequencies and ORs for RCV associated genes are from [29]. RCV effect sizes refer to the excess burden of RCVs in the named gene. CNV and RCV effect sizes are imprecise due to the small number of observations. SNP and PRS data are from [12]. PRS ORs are given for individuals in the top centile relative to all other individuals, and top centile versus the bottom centile. Data for European, East Asian and African American genetic ancestry are given separately. The effect size in the Latino population is not plotted as it has not yet been estimated in sufficient samples. It should be noted that most rare alleles are not expected to confer large effects. The shape of the curve provides an indication of the maximum frequency that selection pressures permit alleles of a given effect size to attain, not the expected effect size for an allele of a particular frequency.

**Fig. 2:**
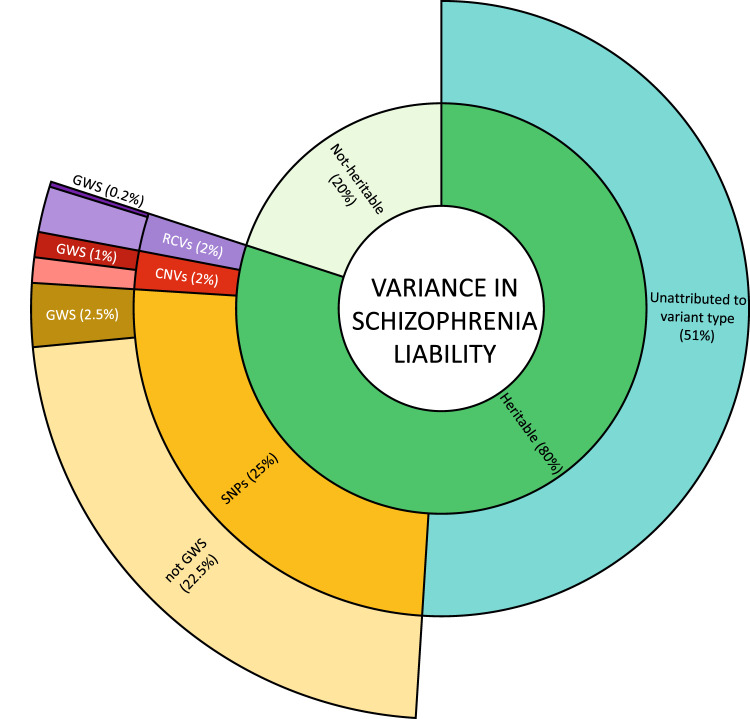
Components of variance in liability to schizophrenia. Inner ring: heritability is estimated from twin studies at around 80%. The remaining 20% attributed to non-heritable risk factors include environmental risk factors, stochastic effects, and de novo mutations. Middle ring: estimates of the contribution to variance in liability from currently known classes of heritable risk alleles. Outer ring: variance in liability assigned to specific risk alleles, or in the case of RCVs, a burden test of RCVs in associated genes. Percentages refer to variance in total liability and are based on studies of people largely of European biogeographic ancestry. Values are approximations (see text). SNP single nucleotide polymorphisms and small insertion/deletion polymorphisms with minor allele frequencies greater than 0.01. CNV large copy number variants with population frequencies less than 0.01. RCV rare coding variants with frequencies typically less than 0.0001. GWS significance surpassing the relevant thresholds allowing for multiple testing for SNPs, CNVs, and burden tests of RCVs.

GWAS have identified an important role for common variants (minor allele frequency >1%). Following the first successful GWAS study of schizophrenia, which identified a single locus containing the gene *ZNF804A* [11], multiple waves of GWAS have been reported, each building on and both confirming and extending the findings of earlier iterations. The largest published GWAS, to date, which included 76 755 individuals with schizophrenia and 243 649 controls, identified 287 associations, 5 of which map to the X-chromosome, meeting standard criteria for genome-wide significance [12] (Fig. 1). Typical of common variant associations to traits associated with low fecundity, the effect sizes are small (mean OR 1.06; range 1.04–1.23) and together the genome-wide significant loci explain only around 2–3% of variance in liability to the disorder, or about 10% of the total variance estimated to be conferred by common alleles (Fig. 2). This study also found that the common variant genetic architecture of schizophrenia did not differ between males and females, the inference being this class of alleles is unlikely to explain reported sex differences in the epidemiology and course of the disorder [13]. Fine mapping of associated loci to identify credible causal SNPs identified a subset of 120 genes that were prioritized as likely to mediate the associations at some of the loci, only a small minority of which (*N* = 16) were implicated by associated variants that change the sequence of the encoded proteins.

**Fig. 3:**
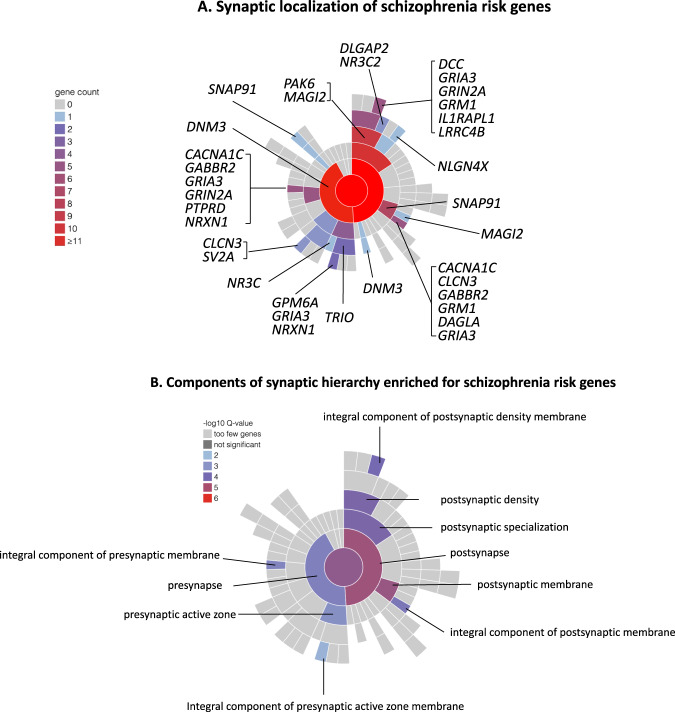
Synaptic location and enrichment of schizophrenia risk genes. **A** Synaptic location of prioritized protein coding genes from Schizophrenia Working group of the Psychiatric Genomics Consortium [12], genes attaining FDR < 0.05 for enrichment for rare coding variants in the study of the Schizophrenia Exome Meta-analysis Consortium [29], and neurexin 1, the only schizophrenia associated CNV to implicate a single gene [20]. Plot and locations were generated and defined according to the Synaptic Gene Ontologies (SYNGO) Consortium Website (https://syngoportal.org). The data required to generate the plot and obtain granular detail of, and the evidence for, the locations and synaptic functions for each gene are available as Supplementary Table 1. Colours denote the number of genes in each cellular component. Numbers are cumulative from the periphery to the centre of the plot respectively depicting the lowest and highest levels of the hierarchy of the ontology. **B** Components of the SYNGO ontology hierarchy are denoted as significantly enriched for genes as in A. Colour denotes the significance of enrichment as determined by SYNGO. Enrichment is calculated relative to a background of all brain expressed genes.

Given the small fraction of common variant liability explained by alleles achieving genome-wide significance, large numbers of common variants remain to be discovered. How many is not resolved, but schizophrenia and other psychiatric, cognitive and behavioural traits are amongst the most polygenic of all human traits [14] with a lower bound from recent estimates of around 10,000 causal variants [15], although other estimates are considerably higher [14]. Consistent with high polygenicity, common risk variants are found in proximity to a very large number of genes, but this is not random. Thus, associations are enriched around genes that are conserved across species, and which are relatively intolerant of mutations in humans [16]. They are also enriched in genes that are expressed in the brain, in neurons, both excitatory and inhibitory, and in genes encoding proteins involved in fundamental biological processes related to neuronal function, in particular gene-sets related to synaptic structure and function (Fig. 3) [12]. Finally, they are also enriched in genes implicated by rare variant studies in neurodevelopmental disorders including schizophrenia [12]. The implications of these patterns of biological enrichment are discussed further below.

#### Rare copy number variants

Numerous studies have consistently demonstrated that rare copy number variations (CNVs), defined as deletions or duplications of DNA segments greater than 1 kilobases (KB) in size, are risk factors for schizophrenia. Across the genome, people with schizophrenia are enriched for rare (<1% frequency) CNVs larger than 20 KB compared with controls, with deletions that overlap genes having the strongest effects on risk [17]. Additionally, the genome-wide rate of de novo CNVs is significantly higher in schizophrenia cases compared with controls [18].

The first specific genetic risk factor to be robustly associated with schizophrenia was a 1.5–3 megabase (MB) deletion of 22q11.2 [19], which had previously been found to cause DiGeorge and Velocardiofacial Syndromes. Following this discovery, CNV studies using SNP genotyping array data from over 20,000 cases and 20,000 controls have identified 12 specific CNVs as risk factors for schizophrenia [17, 20] (Fig. 1). 11 of these CNVs affect multiple genes and are recurrent events formed by non-allelic homologous recombination between low copy repeats, which results in similar CNV breakpoints across carriers. The only single gene disrupting CNV that is currently implicated in schizophrenia involves non-recurrent exonic deletions of *NRXN1*. Individually, the 12 schizophrenia-associated CNVs occur in 0.015%–0.64% of cases [21] but confer strong risks for schizophrenia in individual carriers, with estimated odds ratios ranging between 1.8 and 81.2 [20, 21] (Fig. 1). Being rare events, the confidence intervals for these estimates are wide, and there is some evidence from population studies that the point estimates may be overestimated, although it should be noted that even the largest population study [22] includes relatively few schizophrenia cases (*N* = 1704–2590) and is also unable to provide accurate estimates of effect size. It is clear [17, 20] that additional risk CNVs are identifiable through SNP genotyping arrays, but they are likely to be rarer, smaller in size than can be resolved by arrays, or have smaller effect sizes than those currently implicated, and therefore require larger samples for their discovery.

A duplication of 22q11.2, the reciprocal of the risk deletion at this locus, is the only replicated CNV that is enriched in controls compared with cases [17, 23, 24], suggesting a protective effect against schizophrenia (Fig. 1). The protective effects do not, however, extend to other neurodevelopmental disorders, as it is a risk factor for developmental delay and autism spectrum disorders [25]. From the perspective of exploiting the finding for therapeutics, it is clearly important to determine if duplication of the same or distinct specific gene(s) protects against schizophrenia and increases the risk of other neurodevelopmental disorders.

#### Rare coding variants

Exome-sequencing studies have demonstrated that very rare single-nucleotide variants (SNVs) and small insertions and deletions (indels) that alter the amino acid sequences of genes, collectively termed rare coding variants (RCVs), also contribute to schizophrenia liability. While the exome-wide rate of de novo damaging coding variants in schizophrenia is only modestly higher than expected, there is a stronger enrichment of such variants in cases within genes that are intolerant to protein truncating variants (PTVs) in humans, in genes implicated in early-onset neurodevelopmental disorders, and in genes related to glutamatergic postsynaptic proteins [10, 26, 27], Case-control studies have also shown that in people with schizophrenia, these sets of genes are enriched for ultra-rare (occurring in less than 1 in 10,000 people) damaging coding variants [28, 29].

Sequencing studies are currently underpowered to implicate specific schizophrenia RCVs, but they have begun to identify specific genes where the total burden of any such RCV is significantly greater than in controls. The largest exome-sequencing study of schizophrenia to date was performed by the Schizophrenia Exome Sequencing Meta-Analysis (SCHEMA) Consortium; 10 genes were identified as having an exome-wide significant excess of ultra-rare damaging coding variants through meta-analysis of data from 24,248 schizophrenia cases, 97,322 controls, and 3,402 proband-parent trios [29] (Fig. 1). As a group, these genes were enriched in cases for both PTVs and damaging missense variants, with the gene-specific ORs ranging from 3–50, albeit with large confidence intervals. A subsequent study that meta-analysed targeted sequencing data from 161 genes in 11,580 cases and 10,555 controls with data from the SCHEMA consortium identified two additional risk genes at exome -wide significance [30] (Fig. 1).

## Heritability explained and unexplained

Twin studies suggest inherited alleles (as distinct from de novo or somatic mutations) account for about 60–80% of within population variance in liability to schizophrenia [31, 32] but how this heritability is distributed across alleles of the various frequencies, effect sizes, and types has not been precisely delineated. Current understanding **(**Fig. 2**)** suggests alleles *detectable* by GWAS (i.e. SNP heritability) make the biggest single contribution, estimated at around 25% [12]. RCVs are estimated to have a burden heritability of around 2%, primarily from ultra-rare PTVs but also to a degree from damaging missense mutations [33]. Large rare CNVs may contribute a similar amount to that for RCVs [34]. Thus, assuming additivity, the classes of variation that have been studied most intensively collectively explain around 30% of total variance in liability, or around 40% of the expected heritability. It should be stressed the vast majority of explained heritability is attributable to GWAS loci, RCVs, and CNVs that do not meet stringent criteria for significant association, indeed, only around 10% (but see also ancestry section) of explained heritability is attributable to such findings [12, 17, 33]. Thus, while there has been substantial progress in identifying risk alleles for schizophrenia, there is scope for a great deal more, even using the tools widely in use today.

In schizophrenia as in other common disorders and traits, it is unclear what accounts for the substantial gap between the heritability potentially explained by the current genomic data and that expected from classical genetic epidemiology. It seems certain that some of it will be attributable to classes of allele that are not adequately interrogated by current technology, for example rare non-coding alleles, structural variants other than large CNVs, and polymorphic repetitive sequences, both common and rare, which are difficult to tag by linkage disequilibrium (the phenomenon that makes GWAS possible). It is expected that the potential contribution of these classes of variant will be resolved soon with the increasing use of whole genome and long read sequencing technologies. It is also possible that the heritability captured by SNPs and other types of known risk allele is underestimated relative to family studies due to the higher phenotypic and ancestry heterogeneity in large case control studies. Conversely, the narrow sense (additive model) heritability estimated by genetic epidemiology might be inflated by, for example, statistical gene-gene interactions and inadequate control for shared environments [35–37].

## Ancestry effects

Genomic studies of schizophrenia are based predominantly on participants classified as of European biogeographic ancestry. However, large studies of other ancestries have begun to emerge, of which the most informative was a study of 22,778 cases and 35,362 controls of East Asian ancestry [38]. While novel loci were identified, perhaps the most important finding was that the common variant genetic architecture of schizophrenia is essentially identical in East Asians and Europeans. Similar findings have been noted for other complex traits, including ones that, unlike schizophrenia, show very substantial geographical differences in prevalence [39]. Although the findings suggest that the common genetic architecture, and therefore presumably the fundamental biology, is essentially identical in East Asians and Europeans [38], there is a clear imperative to increase diversity in genomic studies as not all the fruits of genomic studies are likely to generalize across populations. For example, polygenic risk score analysis captures around 8% of variance in liability in people classified as (white) European, 7% as Latino, 6% as East Asian, but only 1.5% those considered African American [12]. Given that polygenic risk scoring is likely to play many roles in healthcare [40], it is critical that the Eurocentric bias of studies be rectified if genomics is not to contribute to further widening inequalities in care provision. At the same time, the inclusion of more diverse samples will increase discovery [38, 41], partly because of increased sample sizes but also because, even if risk loci have similar effects in all populations, they are likely to have higher minor allele frequencies in some populations than in others, increasing the power for their detection [39]. Moreover, the inclusion of haplotypes with diverse patterns of LD is expected to improve the precision of localizing GWAS signals to specific causal alleles [38].

## Pleiotropy, heterogeneity and transdiagnostic effects

### Pleiotropy

A striking finding has been the demonstration of moderate to extensive overlap in common risk alleles between psychiatric disorders suggesting significant biological pleiotropy [42–44], albeit estimates of shared risk might be inflated in some instances by assortative mating [45]. There are various reasons why overlapping genetic effects were not unexpected, including evidence from many large-scale family studies that psychiatric phenotypes do not “breed true” (e.g. [32]). It is important to note that the common allele genetic correlation between two cohorts of people with the same psychiatric diagnosis is typically greater than it is between cohorts with different diagnoses [42, 46] suggesting that, while current diagnostic criteria may not define biologically distinct conditions, they do identify groups of cases whose members have, on average, more in common with each other than they do with groups with other psychiatric disorders.

Pleiotropic associations to schizophrenia PRS have been confirmed in large-scale phenome-wide analyses in population-based samples that assessed hundreds of phenotypes [47–49]. Whilst the strongest associations were for other psychiatric conditions, associations were also found for cognitive, psychosocial, and physical health phenotypes.

### Relationship to other psychiatric and neurodevelopmental disorders

The extent to which common variant liability to schizophrenia is shared with another diagnosis is greatest for bipolar disorder, with a genetic correlation of around 0.7 [44], the overlap being stronger between SZ and BDI than BDII [50]. Given the two disorders share many clinical features, it is important to note the genetic correlation between SZ and BD is substantially higher than can be plausibly attributed to diagnostic misclassification [42] or assortative mating [45]. The strong phenotypic and genetic overlaps (amongst other things) between schizophrenia and bipolar disorder argue against regarding the two as entirely distinct syndromes [8, 9], although the imperfect overlaps in liabilities nevertheless suggest there is some biological validity in distinguishing between them.

The pattern of overlapping genetic risk seen for rare alleles is somewhat different, the evidence suggesting schizophrenia has greatest overlaps with childhood onset neurodevelopmental disorders (NDDs), particularly intellectual disability (ID), autism and attention deficit hyperactivity disorder (ADHD), rather than adult-onset psychiatric disorders. Overlaps occur at the level of genes containing rare disruptive mutations [29, 51, 52], as well as at the level of specific risk alleles, including both CNVs and rare disruptive mutations [51, 53]. Finally, genes implicated by GWAS in schizophrenia are enriched for genes associated with rare disruptive mutations in NDDs [12, 29].

### Symptomatic heterogeneity and transdiagnostic effects

The latent structure of symptoms in schizophrenia consists of positive, negative disorganised and affective symptoms as well as cognitive ability [54–57]. Within cases, the severity of negative and of disorganised symptoms is associated with higher PRS for schizophrenia, and with greater familial aggregation of the disorder [55, 57–60]. Perhaps surprisingly, neither PRS nor familial risk for schizophrenia appear to be associated with positive symptoms in individuals with established illness [55, 57, 58, 60]. One possibility is that samples with established schizophrenia show insufficient variance in positive symptoms to detect the effects, and consistent with this, there is evidence positive symptoms are associated with PRS for schizophrenia in people with bipolar disorder [50, 61, 62] and with positive symptoms in a first-episode psychosis sample which included a broad range of psychosis diagnoses, only around a third meeting criteria for schizophrenia [63].

There is evidence that in people with schizophrenia, the presence of manic symptoms is associated with the burden of bipolar risk alleles carried by an individual rather than liability to schizophrenia per se [56, 59, 64], and it seems likely by extension that similar considerations will apply to depressive symptoms [65]. However, dissecting the molecular genetic underpinnings of affective symptoms in schizophrenia is not straightforward given that some of the liability to those disorders also confers liability to schizophrenia, making it difficult to separate modifier and causal effects. One approach to this is to apply structural equation modelling to try to distinguish between liability that is shared across disorders and liability that that is relatively specific to one disorder [66]. Such methods are yet to be applied to large well-phenotyped schizophrenia samples, but when applied to bipolar disorder, the findings suggest that manic, psychotic (independent of mood), and depressive symptoms are respectively associated with the specific components of liability to BD, schizophrenia and MDD in individual carriers [67]. A picture is therefore beginning to emerge supporting the notion that clinical heterogeneity at least in part reflects aetiological heterogeneity, and that the clinical picture expressed by an individual is the result of a confluence of partly orthogonal symptom dimensions and their underlying genetic risk factors.

### Cognitive impairment

Cognitive impairment is a variable feature of schizophrenia but is strongly and consistently associated with poor functional outcomes [68]. There is a negative genetic correlation between common alleles associated with schizophrenia and those associated with cognitive ability (r_g_ = −0.21) [69], which, given the similar SNP heritabilities of schizophrenia and cognitive ability of 20–25%, implies about 5% of variance in liability to schizophrenia is potentially explained by the shared effects of common alleles on cognition. Schizophrenia PRS have been shown to predict lower cognitive ability in population samples [49, 70]. Within individuals with schizophrenia the evidence is less consistent, some studies finding a negative association between cognitive ability and schizophrenia polygenic risk score [55, 60, 71, 72] but others not [73–75]. CNVs previously associated with schizophrenia have been associated with poorer cognitive ability in population-based samples [76, 77] and in those with schizophrenia [78], as have ultra-rare coding variants [79].

The evidence with respect to premorbid cognitive impairment suggests that, while only a small proportion (10%) of variance is explained by identified genetic risk factors, the majority of this is accounted for by IQ PRS [55, 79, 80]. In contrast, there is little or no effect of schizophrenia common allele liability [55, 81], whereas rare risk alleles are associated with poorer performance [79, 80]. Whether schizophrenia genetic liability is associated with a poor cognitive trajectory or decline after the onset of psychosis is still unclear [55, 60, 82, 83].

### Course and treatment resistance

Phenotypes indicating a more chronic or severe illness course in schizophrenia such as greater number and length of hospital admissions are highly correlated among affected sibling pairs [58] and have been associated with higher schizophrenia polygenic risk scores [12, 84]. Studies have not provided a decisive answer as to whether common variant liability to schizophrenia is elevated in people with treatment resistant schizophrenia (TRS), perhaps due to small samples and heterogeneity in the definition of TRS [85–89], but if it does, the inconsistent findings probably indicate such a link is likely to be fairly small. This conclusion is also supported by what is by far the largest study, which found that, with respect to schizophrenia liability, the common variant genetic architectures of TRS and non-TRS schizophrenia are qualitatively and quantitatively indistinguishable, but that there was an additional contribution of common risk alleles that was relatively specific to people with TRS [90]. Moreover, that relatively specific contribution showed moderately strong genetic correlations with intelligence and cognitive traits, tentatively suggesting TRS might represent a form of the disorder particularly enriched for neurodevelopmental aetiology. Discussed below, rare variants have provided the strongest genetic evidence for a link between schizophrenia and neurodevelopment, but the relationship between these classes of variant and TRS is not yet clear, likely due to the low power of the published CNV and sequencing studies to date. Thus, some have reported a particularly high burden of CNVs in people with TRS [87, 91], but others found no differences [88, 89]. Sequencing studies similarly are not yet conclusive, although in one study, cases with TRS have been reported to be enriched for RCVs in gene sets related to antipsychotic function and to agents involved in the treatment of amoebiasis and other protozoan diseases [92], while in another study, damaging RCVs were enriched in 112 TRS individuals compared with 218 individuals with typical schizophrenia [93]. Neither finding has to our knowledge been replicated in a published manuscript.

## Implications of genetic findings

### The evolutionary paradox of schizophrenia

Schizophrenia is associated with markedly reduced fecundity [94] from which it has been postulated that risk alleles should segregate in the population at very low frequencies due to purifying selection. As expected under a purifying selection model, there is indeed an (approximately) inverse relationship between the effect sizes of risk alleles and their population frequencies (Fig. 1). However, as we have seen, much of the heritability nevertheless comes from common alleles.

Recent studies have cast some light on this “evolutionary paradox” [95]. The population frequency of high penetrance fitness-reducing mutations seems to be determined by mutation-selection-balance whereby risk alleles are selected against, but this is offset by de novo mutations [96]. Evidence to support this has come from schizophrenia risk CNVs [97] which are maintained at higher frequencies than expected from strong purifying selection because they are in mutation hotspots, and therefore rapidly replenished. Individual high penetrance coding variants are, as expected, extremely rare, but the large number of genes involved in schizophrenia offers a sizable genomic target for pathogenic de novo mutation, allowing this class of variant collectively to occur at a higher frequency than might be expected given purifying selection pressures on individual mutations. Finally, regarding common alleles, one popular hypothesis is that these might attain or persist at high frequencies due to pleiotropic effects on traits that confer reproductive advantages to unaffected carriers, a form of balancing positive selection. However, the current evidence suggests that when background selection effects are controlled for, alleles under positive selection are actually depleted rather than enriched for association with schizophrenia [98]. Moreover, while there is evidence that common variant liability to schizophrenia might indeed be associated with pleiotropic effects on increased fecundity, at least in contemporary European environmental contexts, the effects are too small in unaffected carriers to offset the negative impact on the fecundity of cases [99].

Overall, the evidence suggests that purifying rather than positive or balancing selection is the rule for schizophrenia risk variants [98, 100], but at those detected by GWAS, the effects are weak, allowing risk alleles to achieve high frequencies under a mutation-selection-drift model, higher frequencies being facilitated at some loci by a reduction in haplotype diversity due to background selection [96, 98]. These findings do not, however, exclude a role for positive or balancing selection at some loci [101].

### Pleiotropy and trans-diagnostic genetic effects

There are many important implications of the pattern of overlapping genetic effects observed between psychiatric disorders. We highlight four. First, the genomic data support the widespread view that our systems of diagnostic classification are not optimal for basic or clinical research. This does not mean diagnosis-based research should be completely abandoned as there are no generally agreed alternatives of demonstrable superiority for research or for treatment. However, attempts to reduce heterogeneity, define the corresponding underlying biology, and identify novel strata of clinical utility, will only succeed through the pursuit of complementary approaches that cut across or divide current diagnostic groups (or both). Findings referred to above support approaches based on stratifying patients based on patterns of symptoms or cognitive ability, but there are other credible approaches including stratification by a particular aetiological factor such as a rare mutation, or a particular environmental exposure. However, progress in stratification will require access to genomic data from large samples in which the phenotypes have been measured with greater granularity than has hitherto been the norm for genomic studies [58, 102]. Secondly, the pleiotropy seen for both common and rare risk variants for schizophrenia argues against there being a simple one to one mapping of risk alleles onto psychiatric conditions and related traits. This conclusion is strengthened by emerging evidence that, while genetic overlaps between psychiatric disorders and related traits are extensive, there are few disorder-specific variants, and most risk alleles show mixed direction effects on susceptibility to different outcomes [15]. This suggests that individual susceptibility to specific disorders may reflect the specific constellation and effect sizes of highly pleiotropic variants that contribute generally to the development of psychiatric conditions and related traits rather than a set of disorder specific risk variants. It follows that researchers should be extremely cautious interpreting observational or mechanistic studies, whether in humans or model systems, that seek to infer causal relationships between possible underlying genetics or neurobiology and specific diagnoses or other phenotypic outcomes [43]. Finally, the overlaps in genetic risk between schizophrenia and NDDs point to the need to consider the relationship between them, and it is to this that we will turn next.

### The neurodevelopmental continuum

Schizophrenia has long been considered to result at least in part from disturbances of neurodevelopment [103, 104]. Indeed, there is a modest common variant genetic correlation with both autism (rG = 0.21) and ADHD (rG = 0.17) [105, 106]. However, the findings from rare variant studies for overlap at the genic and mutational level with childhood NDDs reviewed above point to a stronger relationship and an extension of the neurodevelopmental hypothesis of schizophrenia [51]. Further supporting the hypothesis implicating shared aetiology between schizophrenia and neurodevelopmental disorders, as reviewed elsewhere, there is evidence that many of the environmental risk factors for schizophrenia impact on the developing brain, and are shared with childhood NDDs [3].

It is important to note that the enrichment of rare risk mutations is not equal across neurodevelopmental disorders, but is greatest in ID, followed respectively by autism and ADHD where the burdens are equivalent [107] and then by by schizophrenia [26, 51]. Moreover, as we discuss above for schizophrenia, but also in ASD and ADHD [107], the burden is higher in those with pre-morbid cognitive impairment. These findings suggest that neurodevelopmental disorders may be conceptualized as lying on a continuum of neurodevelopmental impairment reflecting the relative burden of rare damaging mutations, the magnitude of their effects, and perhaps the timing of their impacts on brain development and resulting functional outcomes [51]. There is also evidence that the phenotype expressed by carriers of the rare mutations that impact on neurodevelopment is influenced by the burden of disorder associated common genetic variants, for example CNV carriers who present with schizophrenia also have an elevated burden of common risk alleles for the disorder [108–110] while rare coding variant carriers with ASD have elevated burdens of common risk alleles for ASD and this associates differentially with the observed phenotypic features [111]. The evidence for a neurodevelopmental continuum points to the need for more transdiagnostic research across neurodevelopmental disorders, which are still largely studied separately, and has implications for nosology and clinical practice [51] as well as basic gene discovery [30, 52, 107]

### What neurobiological mechanisms are implicated by genetics?

It is often assumed that schizophrenia results from pathophysiological perturbations to specific brain regions or circuits. There is strong evidence implicating disturbances of dopaminergic neurotransmission in the genesis of psychotic symptoms, but these are unlikely to explain all the clinical features of the disorder [112]. Moreover, rather than highlighting circumscribed anatomical or functional abnormalities, the hundreds of neuropathological and neuroimaging studies to date point to widespread and variable involvement of many brain regions and circuits [3]. This lack of neuroanatomical specificity has been supported by genomic studies of all classes of genetic variation, both common and rare which, while providing some support for the involvement of dopaminergic neurotransmission [113], have found that genes with high relative expression in most regions of the human brain are enriched for risk variants [12, 29]. Associations are enriched particularly in CNS neurons, both excitatory and inhibitory, and in genes encoding proteins involved in fundamental biological processes related to neuronal function, in particular gene-sets related to synaptic development, maturation, structure and function [27, 29, 114–116] (Fig. 3).

These findings must be interpreted in the context of current limitations in understanding the human brain transcriptome and proteome, regionally and developmentally, the fact that very few GWAS and CNV associations can be robustly linked to specific genes, and that there are errors of omission and commission in assigning biological functions to genes. The latter point is well illustrated by the finding that, even among the genes implicated with high certainty through the precision of exome sequencing, only a minority can be confidently assigned to functions likely to be relevant to schizophrenia. However, as they stand, the recent findings pose an alternative to the view that schizophrenia is the result of dysfunction in a limited set of circumscribed brain regions and circuits. Rather they suggest that fundamentally the condition may best be understood as resulting from disturbances in neuronal, and particularly synaptic, function that are not confined to a small number of brain regions and circuits. Thus, the clinical features of schizophrenia may reflect altered neuronal function across many brain regions and functions, a hypothesis in line with the extreme diversity of psychopathology associated with the disorder and its association with a broad range of cognitive, sensory, perceptual, motor, and other impairments [4–6]. It is also supported by large-scale structural brain imaging studies which have demonstrated reduced brain size and widespread reductions in cortical thickness, surface area, and size of subcortical structures [43, 117] to be associated with schizophrenia, and that a morphometric score representing deviations from the norm from 75 different brain regions could predict both schizophrenia and common variant liability to the disorder [118].

While schizophrenia as a syndrome may result from widely distributed neuronal pathology, it is likely that individual symptoms, cognitive impairments, and other features of schizophrenia are associated with dysfunction in specific brain regions or circuits with the extensive heterogeneity reflecting regional and circuit level variability in the downstream impact of these disturbances in neuronal function. If this is the case, there are important implications for research aiming to identify neurobiological endophenotypes that mediate the effects of genetic risk on behavioural or symptomatic outcomes, and drawing causal inferences may ultimately require experimental validation [43].

On a more positive note, if schizophrenia is essentially a disorder of neuronal, and particularly synaptic, function that is not confined to a specific brain regions and circuits, then it should be possible to gain mechanistic insights from animal and human cellular model systems based on genomic findings. Since models based on high-risk mutations offer the most robust starting points [10], such studies are likely to become a major focus of research efforts over the coming decade. However, researchers will need to keep in mind that the extensive pleiotropy of that class of mutation implies that what is being modelled is not a specific diagnostic entity, and that just as clinical diagnosis in a carrier depends on the common (and no doubt rare) variant liability to a range of other disorders, so will some of the model system phenotypic readouts. Moreover, from the schizophrenia perspective, the use of such variants is likely to bias towards modelling aberrant neurodevelopment as opposed to the other pathophysiological processes that are undoubtedly in play, and that *potentially*, may be more open to remediation.

## Conclusions

In many ways, the past 15 years have seen substantial progress in understanding the genetics of schizophrenia that has yielded more insights than any other area of biological psychiatry. Many specific genes and loci have been implicated and this has begun to point towards some of the neurobiological mechanisms likely to be involved and provided a firm basis upon which mechanistic research can proceed. Genetic findings have revealed the nature of schizophrenia’s close relationship to other conditions, particularly BD and childhood NDDs and provided an explanation for how common risk alleles persist in the population in the face of reduced fecundity.

Yet, as is so often the case in science, with advance has come a greater appreciation of the challenges ahead. Current genomic strategies only potentially explain around 40% of the heritability with a much smaller proportion explained by robustly identified loci. The extreme polygenicity of schizophrenia, together with the implication of many alleles of small effect, pose challenges for attempts to understand biological mechanisms. The high degree of pleiotropy points to the need for more transdiagnostic research and the shortcomings of current diagnostic criteria as a means of delineating biologically distinct strata. It also poses challenges for inferring causality in observational and experimental studies in both humans and model systems. Finally, the Eurocentric bias of genomic studies needs to be rectified to maximise benefits and ensure these are felt across diverse communities.

Many of these challenges can be overcome by the application of new and emerging technologies, such as whole-genome and long-read sequencing, to large and diverse samples. Substantive progress in biological understanding of schizophrenia will require parallel advances in functional genomics and proteomics applied to the brain across developmental stages. However, it is our view that these efforts will only succeed in their ultimate aim of identifying disease mechanisms and defining novel strata if the increased granularity of genomic data can be combined with sufficiently granular phenotypic data. Such phenotypic data should include measures of symptom domains, as well as markers of clinical and functional outcome, all of which have the benefit that they can be applied in transdiagnostic analyses. In addition, the inclusion of candidate biomarkers, such as cognition, neuroimaging or blood-based assays, will help elucidate aetiological pathways between genetic risk and these phenotypic outcomes and have the potential for use in mechanistically informed stratification.

### Supplementary information

Supplementary Table 1

## Data Availability

No new data were generated for this article.
